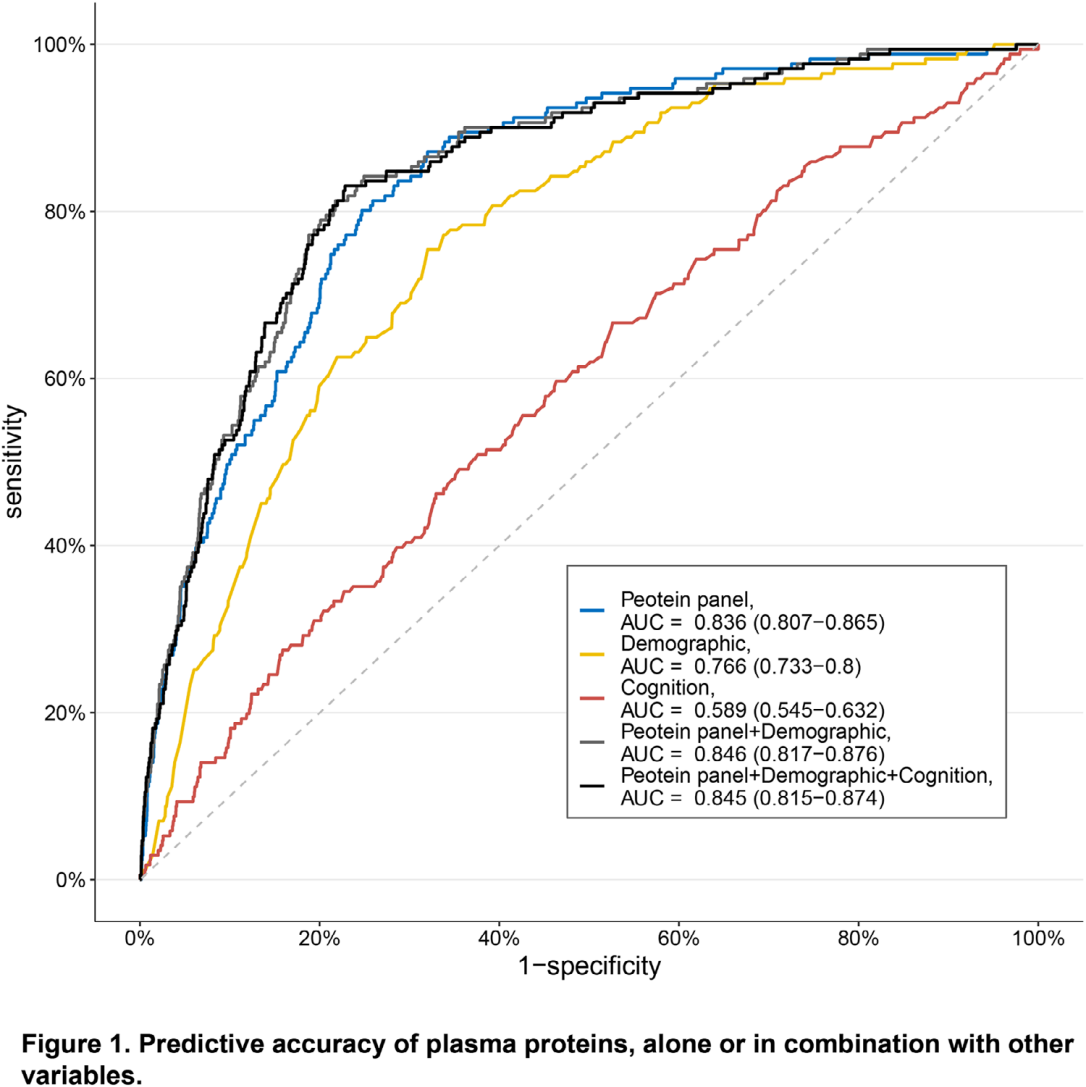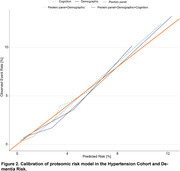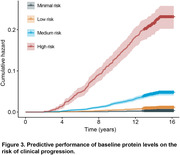# Proteomic dementia risk assessment in hypertension

**DOI:** 10.1002/alz.088906

**Published:** 2025-01-09

**Authors:** Yuye Ning, Zhibo Wang, Meilin Chen, Jianping Jia

**Affiliations:** ^1^ Xuanwu Hospital, Capital Medical University, Beijing, Beijing China; ^2^ Innovation Center for Neurological Disorders, Xuanwu Hospital, Capital Medical University, Beijing, Beijing China

## Abstract

**Background:**

Hypertension is widely prevalent and independently increases the risk of dementia. Advances in high‐throughput plasma proteomics analyzed may offer new opportunities to improve risk stratification for these patients.

**Method:**

This study involved a proteomic analysis of plasma samples collected during the baseline recruitment of participants in the UK Biobank. Participants included in the study were those with hypertension at baseline and no history of dementia, involving approximately 3,000 unique proteins. The primary endpoint of the study was the first occurrence of dementia. The entire cohort was divided into two subsets for feature selection: 80% of the data was used for training and 20% for testing. Feature selection was conducted using least absolute shrinkage and selection operator (LASSO) regression, followed by the development of a predictive model using Generalized Linear Models. This model was then tested within the internal cohort and compared against existing clinical risk models.

**Result:**

This study included 14,038 hypertensive patients without dementia at baseline, with an average age of 59.94 (SD 7.06) years, of which 7,552 (53.8%) were male. During a median follow‐up of 14.4 years, 575 cases (4.1%) of dementia were identified. From the training set, using LASSO regression selected 61 proteins to constitute the primary proteomic risk model (out of a total of 2,924 measured proteins). The Area Under the Curve (AUC) for predicting dementia events using the protein model was 0.836 (95% CI 0.807–0.865). In comparison, the AUC for the demographic model (Age, sex, education, and Apolipoprotein E) was 0.766 (95% CI 0.733–0.8), and the AUC for the cognitive model was 0.589 (95% CI 0.545–0.632). The combination of models yielded an AUC of 0.845 (95% CI 0.815–0.874). Additionally, the primary 61‐protein model demonstrated good calibration (Figure 2). When the entire cohort was stratified by quartiles of predicted concentration according to the model, higher predicted values were associated with increased risk of dementia (Figure 3).

**Conclusion:**

This study has conducted a large‐scale proteomic study on the risk of dementia events in a hypertensive population. The proteomic risk prediction model offers a broad range of dynamic risk stratification and significantly outperforms common clinical feature models.